# Artificial Anus

**Published:** 1843-04

**Authors:** 


### ARTIFICIAL ANUS.

We will not pay our readers the poor compliment to suppose that they have *not* read the learned and valuable paper of Dr. Gross, on *Wounds of the Intestines,* which occupied so large a portion of the three previous numbers. They will, no doubt, be pleased to learn that we shall publish in the Journal for May or June, an article from the same pen on the nature and treatment of *Artificial Anus.* This will be in some sort an appendix to the paper just mentioned, and the two together will form one of the most complete monographs that we know. C.

